# Structural Stability of Optofluidic Nanostructures in Flow-Through Operation

**DOI:** 10.3390/mi11040373

**Published:** 2020-04-02

**Authors:** Yazan Bdour, Juan Gomez-Cruz, Carlos Escobedo

**Affiliations:** 1 Department of Chemical Engineering, Queen’s University, Kingston, ON K7L 3N6, Canada; 16yb6@queensu.ca (Y.B.); 17jmgc@queensu.ca (J.G.-C.); 2 Instituto de Ciencias Aplicadas y Tecnología (ICAT), Universidad Nacional Autónoma de México (UNAM), Ciudad de México 04510, Mexico

**Keywords:** optofluidic, sensor, surface plasmon resonance, nanohole array, mechanical properties, nanofluidic, nanoplasmonic

## Abstract

Optofluidic sensors based on periodic arrays of subwavelength apertures that support surface plasmon resonance can be employed as both optical sensors and nanofluidic structures. In flow-through operation, the nanoapertures experience pressure differences across the substrate in which they are fabricated, which imposes the risk for structural failure. This work presents an investigation of the deflection and structural stability of nanohole array-based optofluidic sensors operating in flow-through mode. The analysis was approached using experiments, simulations via finite element method, and established theoretical models. The results depict that certain areas of the sensor deflect under pressure, with some regions suffering high mechanical stress. The offset in the deflection values between theoretical models and actual experimental values is overturned when only the effective area of the substrate, of 450 µm, is considered. Experimental, theoretical, and simulation results suggest that the periodic nanostructures can safely operate under trans-membrane pressures of up to 20 psi, which induce deflections of up to ~20 μm.

## 1. Introduction

The development of new point-of-care (POC) diagnostic technologies requires low-cost, fully integrated sensing platforms capable of providing quantitative results in situ. At the same time, POC diagnostic platforms have a tremendous potential that is yet to be fully exploited. Telemedicine, for instance, aims to monitor the health of patients remotely through on-site sensing using personal devices, holding a global market of ca. $20 billion USD (United States dollars) [1]. A trendy and increasingly demanded approach to in situ sensing is the use of lab-on-a-chip platforms enabled by cell phones to record, analyze, and transmit the results [2,3,4]. With the recent emergence of new pathogens, such as the Coronavirus and the Yaravirus, an on-site analysis will limit their health impact with a rapid sensing test, quantifying the severity of the infection, and assisting with the quarantine measures [5,6]. Periodic arrays of subwavelength structures fabricated in metal films enable surface plasmon resonance (SPR), which motivated their use as biosensors for several applications in different fields [7,8,9,10,11,12]. Ordered arrays of metallic nanoholes are optofludic structures that enable transport of both fluid and analyte via nanofluidic confinement and nanoplasmonic sensor. The plasmonic resonance signature obtained from nanohole arrays (NHAs) allows the detection of biologically relevant analytes in label-free fashion and real time. Toward the development of POC biosensing platforms, these optofluidic nanostructures are integrated into microfluidic environments in order to create fully integrated sensors compatible with portable electronics [13]. NHA-based sensors are ideal for field applications due to their small footprint and integration abilities as evidenced by recent demonstrations for the detection of bacteria, such as *Chlamydia trachomatis* [14], viruses, such as Ebola [15], cancer biomarkers [16] and uropathogenic bacteria [17]. Flow-through optofluidic structures also enable the enrichment of analytes in liquids by an electrohydrodynamic effect occurring around the NHAs when an electric potential and a pressure bias are applied to the fluid in a closed system [18]. Despite their demonstrated potential in sensing, most applications involving nanohole arrays focus on exploiting the conventional optical capacities of these nanostructures. The mechanical stability of the nanohole membranes is an overlooked aspect of their properties that are key when functioning as nanofluidic structures. In analogy to porous silicon-based membranes, where permeability increases significantly as membrane thickness decreases, the volumetric flow across nanostructured optofluidic sensors increases with the open pore fraction. However, plasmonic nanostructures with built-in thin membranes may suffer from low mechanical stability which could limit, critically, their use as optofluidic flow-through sensors [19,20,21]. The membrane’s mechanical properties change due to the change in the structural morphology of the porous membrane as it deflects under pressure. The stability decreases by a correction factor (1 − *P*), where *P* is related to the porosity of the membrane [22].

Recent studies demonstrate that through-nanoapertures fabricated in thin (~50 nm) gold-coated Si_3_N_4_ substrates offer additional fluidic abilities that can be used to target in-hole delivery of analytes when operated as optofluidic sensors [23,24]. However, flow-through operation results in transmembrane pressures that could potentially damage the rather brittle nanostructures. The mechanical properties of the organized nanohole arrays are not completely understood due to their sensitivity, brittleness, and nano-sized structures. Here, we present a study on structural aspects of Au-on-nitride optofluidic nanoplasmonic sensors operating in flow-through fashion at flowrates compatible with biosensing applications. 

## 2. Materials and Methods

### 2.1. Fabrication of Periodic through Subwavelength Apertures

Through-nanohole arrays were fabricated using focused ion beam (FIB) milling using 100-nm-thick Si_3_N_4_ free-standing membranes (Norcada, Edmonton, AB, Canada) coated with a thermally evaporated 100 nm layer of gold via a 5-nm chromium adhesion layer. Milling was achieved using a gallium ion beam set at 40 keV with a beam current of ~30 pA, with a typical beam spot size of 10 nm, and the dwell time of the beam at one pixel was set to 20 μs. Two arrays of through-nanohole arrays with an area of 20 μm by 20 μm, diameter of ca. 230 nm, and pitch of 560 nm were fabricated.

### 2.2. Fabrication of Microfluidic Chips

**Figure 1:**
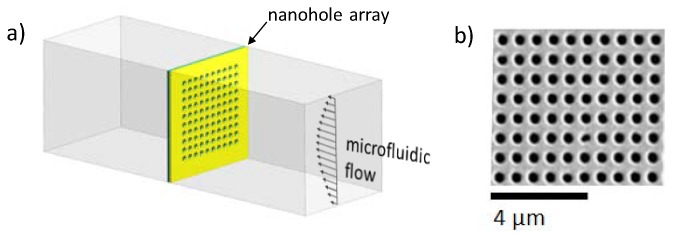
(**a**) SEM image of fabricated periodic subwavelength apertures via focused ion beam (FIB). The nanostructures were 230 nm in diameter and 560 nm in pitch. (**b**) Schematic representation of a nanohole array in a microfluidic chip in flow-through operation.

The microfluidic chip was fabricated using a replica molding technique as described in detail elsewhere [25]. The general steps of the fabrication procedure are briefly described next. A mask with the microfluidic pattern was generated using SolidWorks CAD software (Dassault Systems Solidworks Corp., Waltham, MA, USA). The design included one inlet and one outlet of 1.5 mm, and a 5-mm-wide channel with 100 μm in height. A master was fabricated by spin-coating SU-8 100 photoresist (MicroChem Corp., Newton, MA, USA) on a clean three-inch silicon wafer (Silicon Quest International Inc., Santa Clara, CA, USA). The coated wafer was then prebaked for one minute at 65 °C and for 10 min at 95 °C. The mask with the channel pattern was then placed over the coated wafer and exposed to ultraviolet (UV) light for 90 s. Next, the exposed wafer was hard-baked at 65 °C for 1 min and at 95 °C for 10 min. The master was subsequently developed using a SU-8 developer (MicroChem Corp., Newton, MA, USA). A 12:1 mixture of Sylgard 184 elastomer to curing agent (Dow Corning, Midland, MI, USA) was mixed, degassed in a vacuum, and poured onto the master. After baking at 85 °C for 20 min, the replica was removed from the mold. Inlets and outlets were provided 1-mm punched holes for fluidic access. Microfluidic connections were achieved using polyether ether ketone (PEEK) tubing (Upchurch Scientific, Oak Harbor, WA, USA). A schematic representation of the set-up is shown in Figure 1.

### 2.3. Optofluidic Structure Deflection Analysis

Finite element analysis (FEA) was used as a means to know the order of magnitude of the deflection and the mechanical stress that the optofluidic sensor may experience in flow-through operation. COMSOL Multiphysics (COMSOL, Stockholm, Sweden) was used to simulate a simplified model of the optofluidic sensor under a prescribed unidirectional and orthogonal pressure on one of the faces of the suspended membrane. The simulations were used firstly to estimate the order of magnitude of applied pressures that would result on the deflection of the substrate containing the optofluidic structures. This first model involved a stationary elastic model with default Lagrange–quadratic element type. The finite element analysis solves for the displacement field at a specific point on the membrane for every input force. For the linear model, the system is governed by three tensor partial differential equations: $\nabla \cdot \sigma +F_{v}=0$, $\varepsilon =\frac{1}{2}\left[{\left(\nabla u\right)}^{T}+\nabla u+{\left(\nabla u\right)}^{T}\nabla u\right]$, and C=C(E,v), where σ is the Cauchy stress tensor, $F_{v}$ is the body force per unit, u is the displacement vector, ε is the infinitesimal strain tensor, C is the fourth-order stiffness tensor, E is the Young’s modulus, and v is the Poisson’s ratio. A second static, nonlinear stress–strain model was used to compare the experimental data and to validate the deflection values obtained for the prescribed pressure range. The nonlinear stress–strain behavior was achieved by using a power-law nonlinear elastic material model, accounting for geometric nonlinearities, which is governed by Ludwik’s law, $\tau =\tau_{0}+k\gamma^{1/n}$, where τ is the shear stress, γ is the shear strain, and *n* is an integer [26,27]. A user-controlled mesh with Lagrange–quadratic element type was used for this nonlinear model, to guarantee an acceptable mesh size along the thickness of the modeled substrate. The finite element analysis solves for the displacement field at a specific point on the membrane for every input force. In both models, linear and nonlinear, the parameters of Si_3_N_4_ were mainly used, as the values for the mechanical properties for this material supersede those of the metal components in the sensor, namely, a Young’s modulus of $250\times 10^{9}$ Pa, a density of $3.1\times 10^{3}$ kg/m^3^, and a Poisson’s ratio of 0.23. The surrounding surfaces around the membrane that correspond to the areas that define the thickness of the substrate were set as fixed boundaries. The transmembrane pressures were varied from 1 to 20 psi, as this range corresponds to flow rates on the order of nL/min, which are commonly used in biosensing applications. The deflection of the substrate and the stress (von Mises criterion) were recorded.

In addition to finite element method (FEM)-based models, analytical models on the mechanical behavior of perforated membranes published in the literature were also used to estimate the deflection of the optofluidic sensors in this study, as detailed in the Section 3 [22]. 

## 3. Results and Discussion

**Figure 2:**
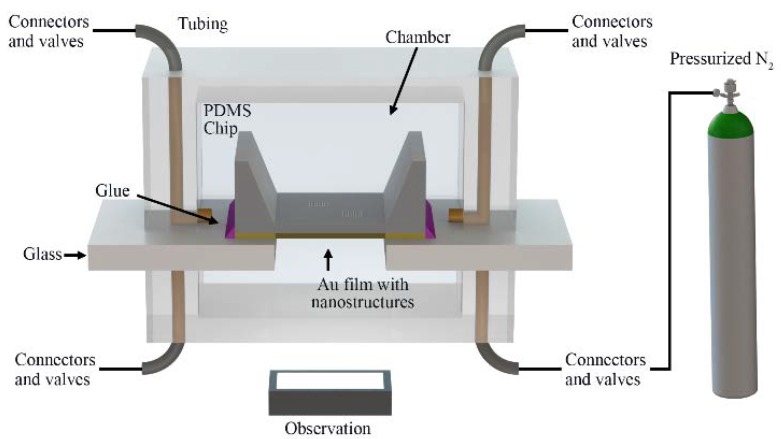
Schematic representation of the experimental set-up.

**Figure 3:**
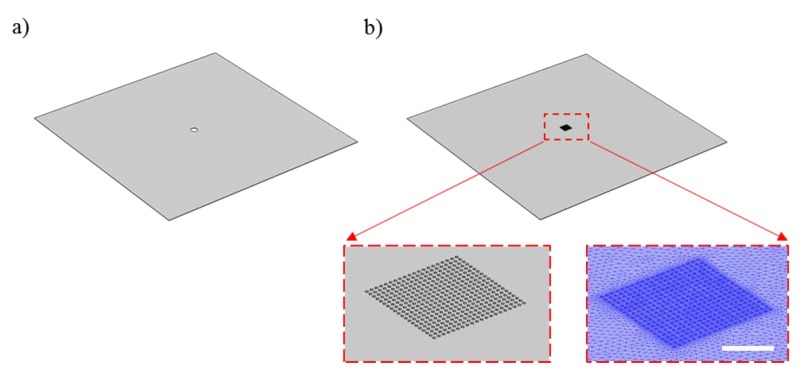
Computer-aided design (CAD) models used for the finite element method (FEM)-based simulations. (**a**) CAD model used for linear elastic simulations. (**b**) CAD model used for the nonlinear elastic simulations. A detail of the nanoapertures in the CAD model and the corresponding mesh are shown as insets. Scale bar represents 10 μm.

Figure 2 shows a schematic representation of the experimental setup used to measure the deflection of the membranes. Figure 3 shows the computer-aided design (CAD) models used to study the deflection of the optofluidic sensors via COMSOL Multiphysics software. Figure 3a shows the simplified model with a single nanoaperture at the center, used in the linear elastic material simulations. The model accounts for a 100-nm-thick membrane with a square surface with a side length of 500 μm, and a circular opening of 10 μm for surface coverage equivalency of the effective surface of the nanoapertures. Figure 3b shows an image of the CAD model used for the nonlinear simulations, a square 100-nm-thick membrane with side length of 500 μm and a 20 μm × 20 μm array of 230-nm-diameter holes with pitch-to-diameter ratio of 2. In both cases, linear and nonlinear models, the mesh curvature factor was 0.6, the maximum element scaling factor was 1.9, the resolution of narrow regions was 0.3, and the optimize quality feature was set to on. The linear model had a maximum element size at all boundaries of $30\times 10^{-9}$. The resulting mesh had ~$210\times 10^{3}$ domain elements with ~$40\times 10^{3}$ boundary elements and ~$1.4\times 10^{3}$ edge elements. The nonlinear model had ~$1.4\times 10^{4}$ domain elements, ~$900\times 10^{3}$ boundary elements, and ~$6\times 10^{3}$ edge elements. The models were solved for pressures applied to the bottom surface of the substrate, for 1 psi, and then using the sweep parameter feature for a pressure range of 2–20 psi with 2-psi pressure increments.

**Figure 4:**
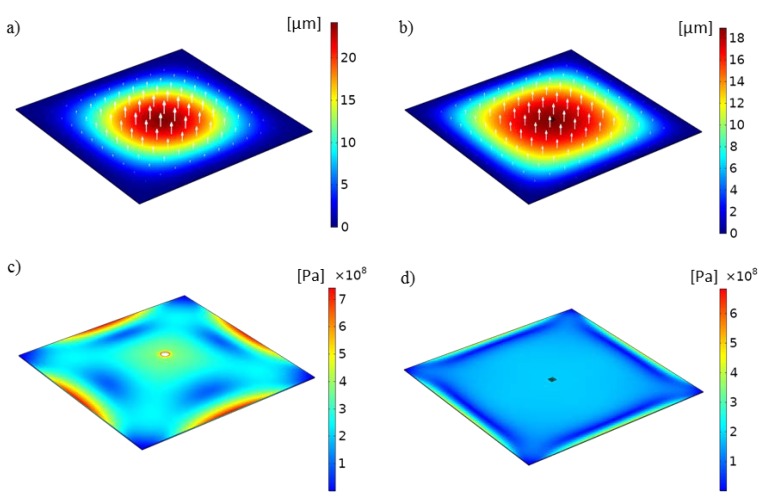
Simulation results of linear and nonlinear models. Membrane deflection under an applied pressure of 20 psi for (**a**) the linear model and (**b**) the nonlinear model. The apertures are shown in the insets within yellow dashed boxes in both cases. Stress distribution (von Mises yield criterion) under an applied pressure of 20 psi for (**c**) the linear model and (**d**) the nonlinear model.

Figure 4 shows images of selected values for the deflection and stress distribution of the model of the membrane under an applied pressure of 20 psi. Figure 4a,b show the displacement in the *z*-direction for the linear and nonlinear models, respectively. The results are presented as non-deformed, with vectors representing the direction and magnitude of the deflection. The pattern of deflection observed from the simulations, as expected, is quasi-circular, with increasing magnitude toward the center of the free-standing membrane. The maximum deflection values, for the linear and nonlinear simulations at an applied pressure of 20 psi, were 24.08 and 19.39 μm, respectively. Maxima were always obtained at the apex of the deformed membrane. Figure 4c,d show the von Mises stress distribution for an applied pressure of 20 psi. The maximum stress found in the simulations was on the order of 1 $\times 10^{8}$ to 10 $\times 10^{8}$ Pa, which suggests that the substrate which is housing the nanoapertures could adequately withstand the deformations resulting from the applied pressure. The simulation results were used to define a range of pressure that could be used experimentally, avoiding failure of the membrane. 

**Figure 5:**
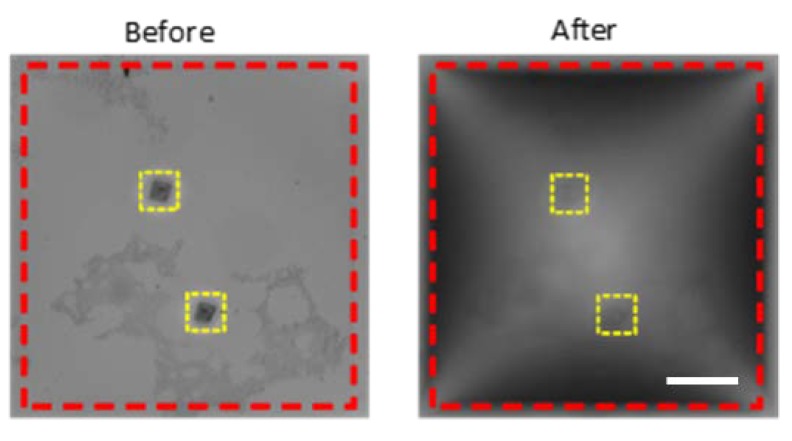
Membrane deflection before and after the application of a pressure of 20 psi. Scale bar represents 100 μm.

Figure 5 shows a bright-field microscopy image of the Au-on-nitride membrane before and after the application of a pressure of 10 psi. The substrate included two rectangular periodic arrays of nanoapertures, indicated with yellow dashed lines. The boundaries of the Si_3_N_4_ membrane are indicated by red dashed lines. The focal plane in both images is the same, which indicates the deflection of the substrate under the applied pressure.

In order to measure the deflection experimentally, the elevation difference at the apex of the membrane was used as reference, and the in-focus *z*-positions were recorded. The applied pressure on the surface of the substrate was monitored and regulated to achieve a constant value throughout the measurement of the deflection. Fringe patterns can be observed in the deflected membrane case, which correspond to the reflected light, confirming a level gradient along the surface of the substrate, and a maximum translation at the apex. The *z*-positioning precision of the inverted microscopy system used in this study was 0.2 µm, which allowed measuring deflections with micrometer precision, at 2-µm intervals. 

**Figure 6:**
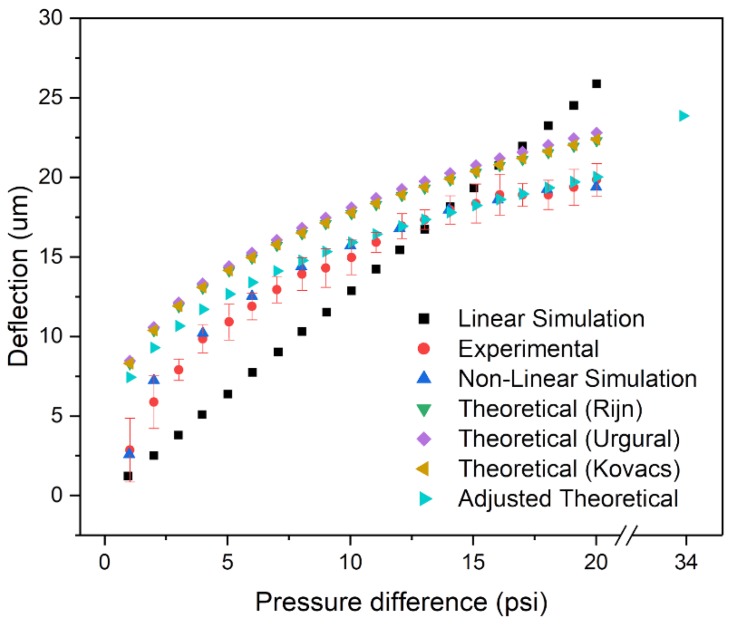
Experimental, theoretical, and simulation results of the maximum membrane deflection (apex). Error bars indicate standard deviation (*n* = 5).

Figure 6 shows experimental and simulations results for applied pressures of 1–20 psi. The trend from the linear simulation model was linear, as expected, with corresponding minimum and maximum deflections of 1.209 and 24.08 μm. In contrast, the deflection results from the nonlinear model decreased with the applied pressure, with minimum and maximum values of 2.584 and 19.39 μm. The same trend was found for experimental values, with the magnitude of the maximum deflection at the apex decreasing with the applied pressure. This can be explained by considering the physical restriction along the frame of the free-standing membrane and due to the mechanical properties of the material. The figure also shows the results from three analytical models that were used to obtain theoretical values, i.e., the Rijn et al. [22], Ugural [28], and Kovacs et al. [29] models, as well as an adjusted Kovacs model fit with the experimental values. These models are similar to each other, whereby they all consider the perforation in a membrane as an error factor affecting the Young’s modulus of the membrane. The deflection of a membrane is given by Equation (1) [30].

(1) $$w_{max}=k_{0}L\;\sqrt[{3}]{\frac{P_{0}\;L}{E_{eff}\;h}},$$

where w is the z-axis displacement, L and h are the size and the thickness of the membrane, and $P_{0}$ is the applied pressure. The constant $k_{0}$ is equal to 0.318, 0.325, and 0.319 within Rijn’s, Ugural’s, and Kovacs’ models, respectively [30]. $E_{eff}$ is the effective Young’s modulus, calculated as $E_{eff}=\left(1-P\right)E_{closed}$, where $E_{closed}$ is the Young’s modulus of unperforated membrane, and P is the correction factor. P is dependent on the perforation and is defined as the fraction of the open areas over the total area of the membrane. As the models are similar, there is negligible difference between the deflection values obtained using the three different models [30]. In the case of the optofluidic sensor, the deformable section of the membrane is smaller than the 500 μm by 500 μm of the free-standing substrate, as observed in Figure 5. The theoretical models do not consider the frame around the deformable area. Therefore, there is an offset between the deflection values obtained using the models and those obtained experimentally, as shown in Figure 6.

The experimental results have a similar trend compared to the theoretical models. Over the non-linear region (<7 psi), the experimental values are on average ~32% below the theoretical maximum, and ~12% below the theoretical maximum within the linear region (>7 psi). The slopes for the experimental and theoretical (Kovacs) values were 0.4831 and 0.4826 μm/psi, respectively within the linear region, with *R^2^* (coefficient of determination (COD)) values of 0.937 and 0.993, respectively. The slopes indicate that the models do not quantify the actual deflection of the membrane. However, they accurately represent the trend of the membrane’s deflection. As such, the unperforated area around the nanohole arrays is influential on the mechanical stability of the membrane. With the assumption that some length of area around the unperforated area does not deflect, then the deflection of the membrane can be rewritten as follows:

(2) $$w_{max,}=k_{0}L_{eff}\;\sqrt[{3}]{\frac{P_{0}\;L_{eff}}{E_{eff}\;h}},$$

where $L_{eff}$, is the effective length of the membrane based on the experimental values, calculated as $L_{eff}=\sqrt{Area\;of\;holes/P_{eff}}$. $P_{eff}$ is the effective correction factor based on the experimental values, where it is assumed that some length around the unperforated area does not deflect. $E_{eff}$ is adjusted to the experimental values and calculated as $E_{eff}=\left(1-P_{eff}\right)E_{closed}$. The model found a range of $P_{eff}$ values based on each experimental deflection point from 2.138 × 10^−3^ to 5.09 × 10^−4^, corresponding to effective membrane lengths of 225 µm to 460 µm, respectively. Figure 6 illustrates that the model is incapable of fitting all the experimental values with one value of $P_{eff}$. The initial deflection value of the experimental values has an $L_{eff}$ of 225 µm, where the $L_{eff}$ non-linearly increases until it plateaus to a constant value of 460 µm within the linear region of the experimental values. The effective length paints a clear image of the membrane’s behavior under pressure. Initially, at low pressures, only the center area of the membrane deflects, while the majority of the membrane is not affected by the applied pressures. As the applied pressure increases, the deflected area grows until it reaches a maximum constant value (460 µm). Even at the maximum value of effective lengths, some outer areas of the membrane do not deflect, reassuring the limitations of deflection model. The experiment was not designed to bring the substrate to mechanical failure; however, the pressure value for the breaking point can be extrapolated from the theoretical model based on the material’s properties. The inflection point of the membrane is not at the edges of the membrane but limited to the effective length of the membrane (i.e., $\;L_{eff})$. Based on the Rijn et al. and Timoshenko et al. models, the maximum pressure applied can be found based on the total stress of the material as shown in Equation (3) [22,31].

(3) $$\sigma_{total}=\sigma_{tensile}+\sigma_{bend}=\frac{0.297}{1-v}\left(1+\frac{1.439}{0.358}\;\right)\sqrt[{3}]{\frac{{P_{0}}^{2}\;{L_{eff}}^{2}\;E_{eff}}{\left(1-v^{2}\right)\;h^{2}}},$$

where $\sigma_{total}$ is the total stress of the membrane, and $\sigma_{tensile}$ and $\sigma_{bend}$ are the tensile stress due to stretching and the maximum bending stress near the middle of the membrane’s deflection edges, respectively. The model is valid when the substrate is under a substantial load that results in large deflections (i.e., $w_{max}/h\gg 1$). Considering that the reported ultimate stress, $\sigma_{ultimate}$, is on the order of 10^9^ Pa, and the intrinsic tensile stress is 10^8^ Pa for a silicon nitride membrane, then the internal stresses can be neglected since they are an order of magnitude lower than the total stress [22]. For a nonductile inorganic material, the $\sigma_{ultimate}$ is equivalent to its yield stress. Taking 2.5 GPa as $\sigma_{total}$, based on the mechanical properties of the material, a pressure of 33.91 psi and deflection of 23.87 μm are obtained, corresponding to the maximum possible values at the verge of mechanical failure [32]. This theoretical maximum deflection value at the verge of failure, which adequately follows the trend of the adjusted theoretical curve, is shown in Figure 6.

## 4. Conclusions

This work presented an investigation of the deflection and structural stability of optofluidic nanohole array-based sensors operating in flow-through mode. The study was approached using experiments, theoretical models, and FEA via computer simulations through FEM. Linear and nonlinear material models were simulated using COMSOL Multiphysics software. The simplified linear model had an expected discrepancy with experimental values, but these were useful to obtain an estimation of the order of magnitude of transmembrane pressures that would allow studying the deflection of the substrate when used in flow-through operation, while avoiding mechanical failure. The discrepancies were up to ~20%. In contrast, the nonlinear model, accounting for a complete nanohole array, accurately described the deflection values obtained experimentally. The stresses corresponding to these deflections can be used to predict maximum operation values that could prevent failure of the optofluidic nanostructures. Three analytical models were used to analyze the deformation of the sensor. The models depicted the behavior of the deflected substrate under pressure but did not intrinsically fit the experimental results since only a fraction of the surface deflects due to the attachment of the free-standing substrate to the silicon frame. Even when the entire 500-µm membrane is under pressure, only a reduced square area, ranging from 225 µm to a maximum of 460 µm per side, deflects. Once adjusted, the theoretical model better fit the experimental deflection values. Based on the models, the fracture point was extrapolated from the maximum yield stress of silicon nitride membranes. As the membranes are composed of nonductile, inorganic material, their yield stress is equivalent their ultimate stress, which resulted with a maximum possible deflection of 23.9 μm, with the applied pressure of 33.9 psi. Although the optofluidic structures are limited by their fragile mechanical stability in flow-through operation, these results show that they are capable of withstanding transmembrane pressures compatible with sensing applications, where the analyte is required to be brought into the apertures. Simulations that could predict the deflection of the structures would greatly benefit the design needs of flow-through optofluidic platforms for specific applications in the context of biosensing.
